# Persistent Microfilaremia Caused by *Dirofilaria asiatica* Infection in 2-Year-Old Child, Sri Lanka, 2024

**DOI:** 10.3201/eid3211.260739

**Published:** 2026-11

**Authors:** Ushani Atapattu, Indeewarie E. Gunaratna, Lucas G. Huggins, Murali Vallipuranathan, Lakmini Liyanage, Harsha K. Narammalage, Nipuni Shilpeswarage, Udayi B. Gunawardana, S.T.A. Prasanga Serasinghe, Vito Colella

**Affiliations:** The University of Melbourne, Melbourne, Victoria, Australia (U. Atapattu, L.G. Huggins, V. Colella); Government of Sri Lanka Ministry of Health, Colombo, Sri Lanka (I.E. Gunaratna, M. Vallipuranathan, L. Liyanage, H.K. Narammalage, N. Shilpeswarage, U.B. Gunawardana, S.T.A.P. Serasinghe); Government of Sri Lanka Regional Director of Health Services, Kalutara, Sri Lanka (H.K. Narammalage, U.B. Gunawardana, S.T.A.P. Serasinghe)

**Keywords:** Dirofilaria asiatica, microfilaremia, parasites, zoonoses, lymphatic filariasis, vector-borne infections, Sri Lanka

## Abstract

Filarial nematodes cause significant illness worldwide, including lymphatic and subcutaneous filariases in tropical regions. *Dirofilaria asiatica* is a recently described canine filarioid increasingly implicated in zoonotic human infections. Here, we report a patent infection in a child in Sri Lanka who had persistent microfilaraemia after 2 rounds of diethylcarbamazine treatment.

Filarial worms can cause significant disease and profoundly affect quality of life for both humans and animals. Key human-infecting species encompass those causing lymphatic filariasis (*Wuchereria bancrofti* and *Brugia* spp.) and subcutaneous and ocular filariases (*Onchocerca volvulus* and *Loa loa*) (*1*). Despite considerable progress in controlling those parasitic diseases, the World Health Organization (WHO) estimates that >650 million persons remain at risk for lymphatic filariasis (*1*). As research into filarial worm control continues, the role that zoonotic species might play in hampering elimination efforts has become more prominent. 

In Sri Lanka, *Dirofilaria asiatica* (formerly known as the *Dirofilaria* Hong Kong genotype) and the *Brugia* Sri Lanka genotype are recognized as 2 zoonotic filarial worms that primarily infect dogs, typically subclinically, and can be transmitted to humans (*2*,*3*). Although knowledge of *D. asiatica* infection in humans is still in its infancy, it has been associated with subcutaneous and subconjunctival nodules and potentially life-threatening anaphylaxis during surgical worm excision (*4*). Furthermore, nodules caused by *D. asiatica* worms can mimic serious conditions (*5*) such as breast tumors (*6*) or testicular torsion, complicating its diagnosis (*7*). Sri Lanka has reported the highest number of human subcutaneous infections caused by *Dirofilaria* spp. worms; those accounted for >50% of subcutaneous nodules in pediatric surgical patients (*8*). The increasing number of reported human cases of *D. asiatica* infection in the region might reflect its important role as a cause of canine subcutaneous dirofilariasis in the Asia-Pacific, potentially increasing opportunities for zoonotic transmission (*3*,*5*,*9*,*10*). Nonetheless, humans are considered dead-end hosts for *Dirofilaria* spp. worms; cases of microfilaremia are rarely observed (*11*). 

We report on a case of *Dirofilaria* spp. infection in a child identified by the Anti-Filariasis Campaign, part of a national postvalidation surveillance program in lymphatic filariasis–endemic districts in Sri Lanka. The Ethics Review Committee of the Postgraduate Institute of Medicine, University of Colombo, Sri Lanka, approved molecular confirmation of positive samples for the program (ID no. ERC/PGIM/2023/133). We obtained informed consent from adults, and parental assent for children, as described previously (*3*). 

## The Study

In April 2024, a 2-year-old girl with trisomy 21 from Kalutara District, Sri Lanka, was confirmed to have microfilaremia caused by an unidentified filarial parasite during routine night blood smear surveillance conducted as part of an ongoing campaign. Methylene blue staining revealed unsheathed microfilariae with a rounded anterior end, 1–2 cephalic nuclei, and a curved tail (Figure), consistent with *Dirofilaria* spp. (*2*). We observed no clinical signs of lymphatic filariasis. We collected a whole-blood sample and administered oral diethylcarbamazine (3 mg/kg/d for 12 d). One month posttreatment, night blood smear showed persistent microfilaremia, prompting a second course of diethylcarbamazine. The patient did not return for care after the second treatment. Attempts to contact the family were unsuccessful until a year later; we then initiated follow-up testing. Microscopy remained positive for microfilariae with morphology consistent with *Dirofilaria* spp., but results for both *Brugia* Test Plus (BT+) (Drugs & Diagnostics for Tropical Diseases, https://www.ddtd.org) and STANDARD Q Filariasis Antigen test (SD Biosensor, https://www.sdbiosensor.com) were negative. Eight days later, we initiated a third 12-day course of diethylcarbamazine (6 mg/kg/d) with albendazole (400 mg). Four weeks after treatment, in July 2025, a thick blood smear specimen tested negative for microfilariae.

**Figure:**
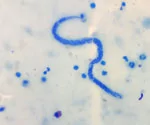
*Dirofilaria asiatica* microfilaria isolated from a 2-year-old girl from Kalutara District, Sri Lanka, stained with methylene blue. The microfilaria has a rounded anterior end with 2 cephalic nuclei (visible at lower right) and a curved tail. Original magnification ×1,000.

We performed molecular characterization on whole blood from the patient, initially collected in 2024, at the University of Melbourne (Melbourne, VIC, Australia). We extracted DNA using the DNeasy Blood and Tissue Kit (QIAGEN, https://www.qiagen.com) and analyzed it using a prevalidated nanopore-based metabarcoding assay capable of detecting all filarial worm species through selective amplification and sequencing of the mitochondrial cytochrome c oxidase 1 (*cox-1*) gene (*12*). We used a second nanopore-based metabarcoding assay targeting the nuclear 18S ribosomal RNA (rRNA) gene, internal transcribed spacer (ITS) 1 and ITS-2 regions, and 28S rRNA gene to characterize the blood microfilariae (*2*) (Appendix). Both assays identified *D. asiatica* worms as the cause of the infection. We identified no other filarial worm infection. We deposited the generated sequences into GenBank (accession nos. PZ167699 and PZ168005).

## Conclusions

We document a confirmed case of persistent patent *D. asiatica* infection in a human. Microfilariae were observed in the patient’s blood over a period of >1 year. Of interest, a small number of human microfilaremia cases have been reported for the closely related filarial species *D. repens*; therefore, its occurrence with *D. asiatica* infection may not be entirely unexpected (*11*). A potential contributing factor could be the patient’s underlying trisomy 21, which impairs both innate and adaptive immunity (*13*); those immunologic vulnerabilities might have enabled the reproductive maturation of *D. asiatica* worms in this young patient.

The ability of *D. asiatica* worms to produce patent infections has public health implications beyond individual cases. In filariasis-endemic settings, the morphologic similarity of *Dirofilaria* microfilariae to those of other known pathogens could complicate microscopy-based surveillance, particularly where species identification relies on field-level morphologic assessment.

This report further highlights the need for surveillance programs to adopt more versatile and species-discriminating diagnostic platforms. For example, recent postvalidation surveillance in Sri Lanka detected *Brugia*-like microfilaremia in 52 persons, including 17 case-patients in Kalutara District; molecular characterization using nanopore metabarcoding of available cases identified *Brugia* SL genotype but found no filarial coinfections (*2*). Such improvements are critical given that the transmission dynamics of *D. asiatica* and *Brugia* SL genotype differ from anthroponotic species; specifically, those species are maintained in canine (*3*,*10*) and potentially feline reservoir hosts. Hence, the control of zoonotic filarial worms such as *D. asiatica* and *Brugia* SL genotype might not be adequately addressed through conventional elimination strategies. For example, despite its benefits in reducing lymphatic filariasis, mass drug administration (MDA) is unlikely to reduce zoonotic transmission of *D. asiatica* worms, particularly given their high (≈33%) prevalence in dogs in the southern and western regions of Sri Lanka (*10*). Moreover, molecular detections of *D. asiatica* worms in dogs have increased across Asia, indicating a broader distribution than previously assumed (*9*,*10*). Emerging evidence suggests that many human dirofilariasis cases in the Asia-Pacific region previously attributed to *D. repens* infection might have actually been caused by *D. asiatica* infection, a misattribution potentially resulting from previous limitations in diagnostic tools and reference data (*2*). In the case we describe, imaging, including ultrasonography, to locate adult worms, could not be performed because we could not reestablish contact with the patient and her family.

Persistent microfilaremia after repeated courses of diethylcarbamazine might reflect treatment failure or reinfection, suggesting inconsistent efficacy against *D. asiatica* and the need for improved therapeutic regimens for zoonotic filarial infections. No established treatment protocols exist for human dirofilariasis with microfilaremia; reported therapeutic approaches, including ivermectin and anti-*Wolbachia* therapy with doxycycline, are largely based on experience with *D. repens* infections rather than controlled studies (*14*). Furthermore, the use of ivermectin in very young children is limited because its safety and effectiveness have not been established in children weighing <15 kg (*15*). 

Our findings demonstrate the ability of *D. asiatica* worms to cause microfilaremia in humans; the clinical implications of patent *D. asiatica* infection remain unknown. This study underscores critical gaps in diagnostic and therapeutic strategies for lymphatic filariasis surveillance and control in regions where cryptic and zoonotic filarial species cocirculate, supporting the need for integrated One Health approaches. Species-specific diagnostic tools within filariasis surveillance will enable accurate detection, which in turn will provide a clearer understanding of the distribution and prevalence of patent zoonotic filarial infections in endemic regions.

Appendix Additional information about persistent microfilaremia caused by zoonotic *Dirofilaria asiatica* infection in 2-year-old child, Sri Lanka, 2024.
