# Supplementary material for: Persistent Microfilaremia Caused by Dirofilaria asiatica Infection in 2-Year-Old Child, Sri Lanka, 2024
Source: Emerg Infect Dis. 2026 Nov;32(11):1890–2. doi: 10.3201/eid3211.260739 (PMC13715880; doi:10.3201/eid3211.260739)
Supplement: Supplementary file 1 [file 26-0739-Techapp-s1.pdf]

Article DOI: https://doi.org/10.3201/eid3211.260739

EID cannot ensure accessibility for supplementary materials supplied by authors. Readers who have difficulty accessing supplementary content should contact the authors for assistance.

Persistent Microfilaremia Caused by *Dirofilaria asiatica* Infection in Child, Sri Lanka, 2024

Appendix

Results of Oxford Nanopore Technologies–based metabarcoding assays in a 2-year-old girl with *Dirofilaria asiatica* microfilaremia

Metabarcoding assay targeting mitochondrial cytochrome *c* oxidase 1 (*cox-1*) gene

- Total reads: 144,595 reads
- High quality reads: 70,346
- *Dirofilaria asiatica* reads of the total high-quality reads (100% identity and query coverage, 668 bp): 69,001 (98.1%)

Consensus sequence generated from nanopore data: NCBI GenBank accession **PP843574**.

Metabarcoding assay targeting the nuclear 3′ 18S ribosomal RNA (rRNA) gene, internal transcribed spacer (ITS)-1, -2, and 5′ 28S rRNA gene

- Total reads: 51,020 reads
- High quality reads: 49,705
- *Dirofilaria asiatica* reads of the total high-quality reads (99.9% identity, 100% query coverage, 1,594 bp bp): 49,705 (100%)
- Consensus sequence generated from nanopore data: NCBI GenBank accession **PX956661**.
